# Deep Learning-Assisted Design for High-Q-Value Dielectric Metasurface Structures

**DOI:** 10.3390/ma18071554

**Published:** 2025-03-29

**Authors:** Junchan Liao, Zhenxiang Shi, Dihang Dou, Haiou Lu, Kai Ni, Qian Zhou, Xiaohao Wang

**Affiliations:** 1 Department of Precision Instrument, Tsinghua University, Beijing 100084, China; liaojc96@163.com; 2 Shenzhen International Graduate School, Tsinghua University, Shenzhen 518055, China; szx21@mails.tsinghua.edu.cn (Z.S.); doudh23@mails.tsinghua.edu.cn (D.D.); ni.kai@sz.tsinghua.edu.cn (K.N.); wang.xiaohao@sz.tsinghua.edu.cn (X.W.); 3 State Key Laboratory of Precision Measurement Technology and Instruments, Department of Precision Instrument, Tsinghua University, Beijing 100084, China

**Keywords:** metasurface, optical sensing, deep learning, spectral

## Abstract

Optical sensing technologies play a crucial role in various fields such as biology, medicine, and food safety by measuring changes in material properties, such as the refractive index, light absorption, and scattering. Dielectric metasurfaces, with their subwavelength-scale geometric features and the ability to achieve high-quality-factor (Q-value) resonances through specific meta-atom designs, offer a new avenue for achieving faster and more sensitive material detection. The resonant wavelength, as one of the key indicators in meta-atom design, is usually determined using traditional solving methods such as electromagnetic simulations, which, although capable of providing high-precision prediction results, suffer from slow computational speed and long processing times. To address this issue, this paper proposes a forward prediction network for the amplitude spectrum of dielectric metasurfaces. Test results demonstrated that the mean square error of this network was consistently less than $10^{-3}$, and the neural network required less than 1 s, indicating its high-precision prediction capability. Furthermore, we employed transfer learning to apply this network to predict the near-infrared transmission spectra of high-Q-value resonant dielectric metasurfaces, achieving significant effectiveness. This method greatly enhanced the efficiency of metasurface design, and the designed network could serve as a universal backbone model for the forward prediction of spectral responses for other types of dielectric metasurfaces.

## 1. Introduction

In recent years, metasurfaces have emerged in various research fields owing to their unique characteristics. Apart from possessing the distinctive electromagnetic and optical properties of conventional metamaterials, metasurfaces also offer advantages such as thin thickness, low loss, ease of fabrication, wide bandwidth, and high transmittance [1,2,3,4]. They show promising applications in super-resolution imaging [5,6], lithography [7,8], and electromagnetic stealth [9,10], among others. The design of metasurface structures is crucial and inherently involves an “inverse problem”. However, due to the complexity of metasurface optical responses, practical design often starts from a “forward problem”, where electromagnetic simulation software calculates the optical responses of meta-atoms under different structural parameters, followed by an exhaustive search to find the parameters that best meet the design requirements. Many mature electromagnetic simulation software tools have excessive computational requirements [11], resulting in high time costs for optimizing metasurface designs. As metasurface structures become increasingly complex in form and arrangement, the optimization dimensions of metasurface structure parameters also increase [12]. Manual efforts alone are insufficient to meet the demands of metasurface optimization design.

To enhance the efficiency of metasurface design, many researchers have begun to widely adopt optimization algorithms. Deep learning, as an efficient category of artificial intelligence technology, has been extensively applied in this field. Several neural network structures have been developed in academia specifically for designing meta-atoms. Christian et al. modeled complex all-dielectric metasurface systems using deep neural networks [13], achieving highly accurate transmission spectra with mean square errors on the order of $10^{-3}$, and computation speeds several orders of magnitude faster than electromagnetic simulation software. Sensong et al. proposed a novel method using neural networks to predict the transmission coefficients of metasurfaces [14], accurately predicting the phase of electromagnetic waves, and the method is generally applicable to the design of various metasurface devices across the entire electromagnetic spectrum. Liu et al. proposed using the ResNets-10 model to predict the $S_{11}$ parameters of plasmonic metasurfaces [15], achieving intensity and phase modulation of metal–insulator–metal metasurfaces, significantly reducing the time required for software simulation processes. Liao et al. proposed a deep neural network to achieve the forward prediction and design of three-dimensional chiral plasmas [16], applying deep learning methods to specific process research on chiral plasma metasurfaces.

Recent advancements demonstrate deep learning’s impact on plasmonic nanostructure design through bidirectional frameworks addressing both forward prediction (optical response from geometry) [17] and inverse design (geometry from target response) [18,19]. Some innovations include multi-task architectures for chiral metamaterials [18], autoencoder-based multi-topology optimization, and iterative multivariable methods resolving non-uniqueness in inverse problems. Deep learning accelerates design workflows by $10\times 10^{5}$ compared to FDTD/FEM simulations while achieving high accuracy [19] and 90% geometry prediction precision [17]. Applications span efficient metasurfaces, structural color engineering, and biomolecular sensing, with demonstrated industrial relevance in high-resolution printing and artwork fabrication. While deep learning eliminates traditional trial-and-error cycles, three systemic limitations persist: (1) the simulation-to-fabrication gap from training on idealized synthetic data (COMSOL/FEM) lacking experimental imperfections [20], (2) geometric rigidity in predefined templates (H-shapes [20], Yin–Yang structures [17]) restricting free-form topology exploration, and (3) interpretability challenges in black-box models obscuring structure response physics. Emerging solutions like physics-informed neural networks (embedding Maxwell’s equations) and hybrid deep learning GAN architectures show promise in balancing data efficiency with physical plausibility [20]. Future progress hinges on integrating real-world fabrication constraints into training loops and expanding to active/tunable systems (phase-change materials and liquid crystals) [18], ultimately establishing a cornerstone in next-gen nanophotonic foundries.

Recent advances demonstrate deep learning’s role in topological photonics, addressing key challenges in modeling, classification, and inverse design [21,22,23,24,25]. Neural networks effectively predict topological invariants (e.g., Berry phase) and optimize photonic structures by learning nonlinear relationships between geometric/temporal parameters and optical responses [24]. Specific applications include classifying edge states in SSH-like photonic lattices using intensity-only measurements [21], circumventing phase retrieval requirements [22], and designing topological photonic time crystals with target momentum-gap properties [24]. Tandem neural networks and cyclic convolutional architectures resolve ill-posed inverse problems by mitigating dimensional mismatches and non-unique mappings in photonic design spaces [23]. While linear methods (e.g., SVM) achieve comparable accuracy for certain tasks, deep learning excels in handling high-dimensional parameter spaces and complex topology–property relationships, as validated through band structure predictions and disorder-robust designs [23]. Deep learning emerges as a versatile toolkit for topological photonics, bridging theoretical topology (e.g., bulk–edge correspondence) with practical device engineering [21]. Its strengths lie in enabling the data-driven inverse design of complex systems where traditional methods fail—particularly in scenarios involving temporal modulation (PTCs) or intensity-only diagnostics. However, limitations persist: reliance on synthetic training data raises questions about experimental robustness, while interpretability gaps hinder physical insights into learned topology–property correlations [21,22]. The field’s next frontier involves scaling these frameworks to 2D/3D systems and multi-band topologies while addressing dataset scarcity through physics-informed training [24]. Crucially, the synergy between deep learning’s pattern recognition capabilities and topological photonics’ robustness principles opens pathways for designing defect-tolerant photonic devices, though experimental validation remains imperative to transition these computational advances to real-world applications.

Although neural networks have made some progress in metasurface design, there are still common issues: the input to the network consists only of parameter data such as the height, radius, and period of the metasurface structure, while the output is much larger than the input parameter quantity, leaving room for further optimization of the network.

Designing a lightweight universal forward prediction network for metasurface optical response is of great significance. This network can be used via transfer learning methods for different downstream-specific functional design tasks, thereby greatly simplifying the metasurface design process and effectively improving the efficiency of metasurface design. Furthermore, this paper addresses the problem of mismatch between neural network inputs and outputs by standardizing and constraining the data representation, enabling the new data structure to represent metasurface structures of different shapes arbitrarily, effectively enhancing the network’s generalization ability. The neural network is also applied to the design of high-Q-value metasurface structures, solving the problem of “spectral line jumping” caused by large resonance peaks in transmission spectrum prediction.

In this paper, we simulated meta-atoms using the finite-difference time-domain method to obtain the transmission spectra of two types of structures: square pillars and elliptical pillars. We used the top view of the metasurface structure as structural data in the dataset. To increase the diversity of the dataset, we performed data augmentation. Based on the dataset of metasurface structure images and transmission spectra, we designed a metasurface forward prediction convolutional neural network and optimized the network structure and training strategy. Based on reducing neural network parameters and improving computational efficiency, we achieved high-precision prediction results with mean square errors reaching below the level of $10^{-3}$ in testing. We also applied transfer learning methods to predict the transmission spectra of resonant metasurfaces in the near-infrared under continuous confined resonance modes, achieving accurate transmission spectra and resonance peaks. Additionally, the deep learning-assisted design method is also applicable to the metasurface of metallic materials [14,15].

Our method proposed in this paper can replace electromagnetic simulation software to efficiently obtain the optical responses of meta-atoms in applications. Furthermore, this network can serve as a universal backbone model to assist in accelerating metasurface design and can be widely used in downstream tasks of different metasurface designs with good generalization effects.

## 2. Methods

Deep learning, as a data-driven algorithm, imposes certain requirements on the quality of the dataset. Therefore, it is essential to acquire a sufficient amount of metasurface data before training. Due to the complexity and high cost of metasurface fabrication processes, experimental data collection is often inadequate to meet the demands of deep learning for large datasets. Thus, this paper utilizes electromagnetic simulation software, specifically FDTD Solution, to simulate various meta-atoms and generate the raw dataset. The simulation software was Ansys Lumiral’s FDTD Solutions (Ansys Lumerical 2020 R2.4).

Common microstructure forms used in all-dielectric metasurfaces based on transmission phase modulation include square pillars, cylindrical pillars, and elliptical pillars. Based on existing research, this study selects square and elliptical pillars as the basic structural types for the dataset. Overtones and combination bands of molecular chemical bond vibrations located in the near-infrared (NIR) are much weaker than the fundamental vibrations. Related research such as the surface-enhanced Raman scattering (SERS) effect and surface-enhanced infrared absorption (SEIRA) have confirmed that the intensities of adsorbates on the nanoparticle substrate or metallic metasurfaces could be intensively amplified. However, the Ohmic loss of metallic metasurfaces has impeded their applications. All-dielectric metasurfaces are realized as high-Q resonators, which are employed to boost up the target bands of overtones. So our current laboratory research is primarily focused on the near-infrared spectrum, and the data collection is conducted within the near-infrared wavelength range. All meta-atoms in this study consist of Si pillars and ${\mathrm{SiO}}_{2}$ substrates, with a unit period of 400 nm, substrate thickness of 250 nm, and Si pillar height of 600 nm. The dimensions of square Si pillars (length and width) and elliptical Si pillars (major and minor axes) are variable parameters, with ranges set at 100 nm to 280 nm for length and width, and 120 nm to 320 nm for the sum of the major and minor axes. The sampling step size during the simulation is set to [value]. A directional polarized plane wave is used as the incident light source, with a wavelength in the near-infrared spectrum, perpendicular to the axis direction of the meta-atom.

Furthermore, to accurately predict the optical response of metasurfaces, continuous meta-atoms on the designed band are selected, and their transmission spectra for directional polarized light are used as labels for the dataset. A total of 361 sets of data for square pillars and 441 sets for elliptical pillars were obtained through simulation software.

However, relying solely on the structural parameters of meta-atoms as inputs limits the predictive capability of the network. Recent research on optimization design algorithms for metasurfaces based on genetic algorithms and convolutional neural networks has proposed using binary encoding of top-down views of metasurfaces to represent their structures. In our analysis, the metasurfaces studied in this paper also conformed to binary encoding standards. Therefore, the metasurface structure data, i.e., the input to the neural network, will also be based on top-down images, and there are two advantages to this. One advantage is that using images as inputs can overcome the problem of mismatch between input and output data dimensions when geometric parameters are used as inputs. The other advantage is that using images as network inputs applies to convolutional neural networks, and arbitrarily shaped structures can be expressed in the same data form.

**Figure 1:**
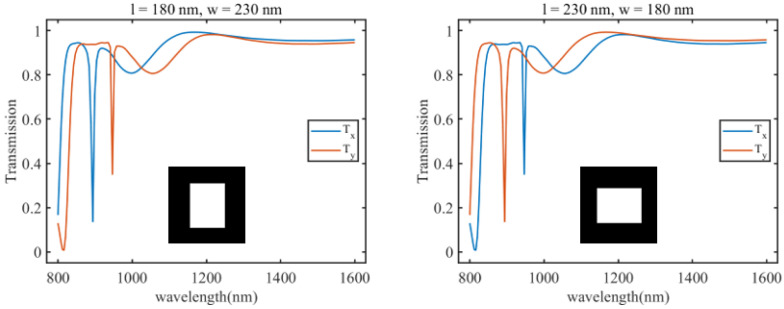
$T_{x}$ and $T_{y}$ of two types of metasurface units with a 90° difference in the rotation angle of the Si pillar.

To further increase the training data quantity, augmentation techniques are applied to the existing data. Currently, in the dataset, both square and elliptical pillar meta-atoms have a rotation angle of 0° within the plane. Therefore, while keeping the dimensions and shapes of the meta-atoms unchanged, the dataset is expanded by varying the rotation angle of the Si pillar structure. Three different rotation angles (θ = 30°, 45°, and 60°) are chosen to obtain transmission spectra $T_{x}$ for *x*-direction polarized light for meta-atoms. During the parameter scanning process, when the major and minor axes of the elliptical pillars are equal, a polarization-independent cylindrical structure is generated, and duplicate results are found in different angle scans. Hence, polarization-independent structures are manually removed from the dataset. Each rotation angle yields 361 sets for square pillars and 420 sets for elliptical pillars. Additionally, as the current dataset only contains transmission spectra $T_{x}$ of meta-atoms for *x*-direction polarized light, to enable the neural network to learn more information, transmission spectra $T_{x}$ for *y*-direction polarized light are added to the dataset, serving as labels in combination with the existing data. As shown in Figure 1, for any given structure, after rotating the Si pillar by 90 degrees, $T_{x}$ and $T_{y}$ will interchange. Therefore, we do not need to perform additional simulation work; instead, during the parameter scanning process, we effectively capture all structures with Si pillars rotated by a 90-degree difference in angle. When the Si pillar rotation angle is 0°, observing the meta-atom where length and width are interchanged, both $T_{y}$ are identical. Thus, by rotating the meta-atom Si pillar angle and increasing $T_{y}$, we have completed data augmentation, resulting in a final dataset of 3145 sets of meta-atom images and transmission spectra.

**Figure 2:**
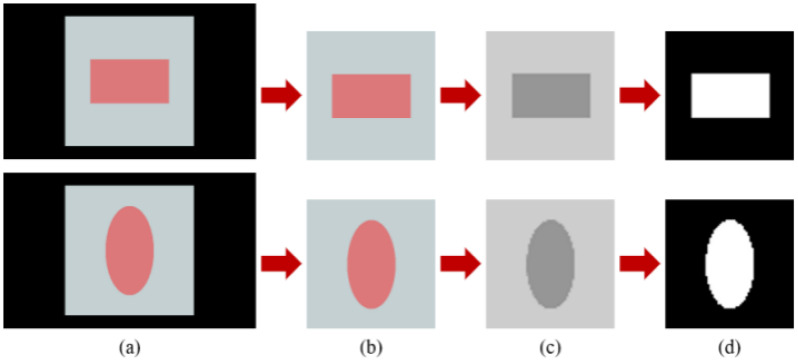
The preprocessing process of meta-atom images. (**a**) Original image; (**b**) removal of non-structural parts; (**c**) conversion to grayscale; and (**d**) image binarization.

Preprocessing the data before training the neural network is necessary. This paper mainly involves four steps: removing non-structural parts, compressing image sizes, converting images to grayscale, and binarizing images. The original images in Figure 2a have black backgrounds for non-structural parts. By thresholding to distinguish between the background and structural parts, the structural parts (Figure 2b) shown in Figure 2a are obtained, and regarding the comprehensive consideration of network computing convenience and resolution, the image dimensions have been determined to be 64 × 64 pixels. Considering that metasurface structural images only consist of ${\mathrm{SiO}}_{2}$ substrates and Si pillar microstructures, the RGB images are converted into grayscale (Figure 2c) with a single channel, effectively reducing the input parameters of the neural network. Finally, the structural images are binarized (Figure 2d), with ${\mathrm{SiO}}_{2}$ substrates assigned a grayscale value of 0 (black) and Si pillars assigned a grayscale value of 255 (white). Binarizing the images further highlights the boundary between the substrate and structure and facilitates normalization before training the neural network. All image preprocessing steps are completed using OpenCV. Regarding OpenCV, it is an open-source computer vision library that provides a number of functions that efficiently implement computer vision algorithms, covering everything from the most basic filtering to advanced object detection. OpenCV is developed in C/C++, and also provides interfaces to other languages such as Python, Java, MATLAB, and so on, making it easy for developers to use OpenCV’s features. All experiments in this study were conducted on our laboratory server, which has 4 NVIDIAGeForceRTX2080Ti graphics cards, 8 CPU cores, and a total of 64 GB of graphics memory and 1024 GB of storage space, with a CUDA version of 11.6.

**Figure 3:**
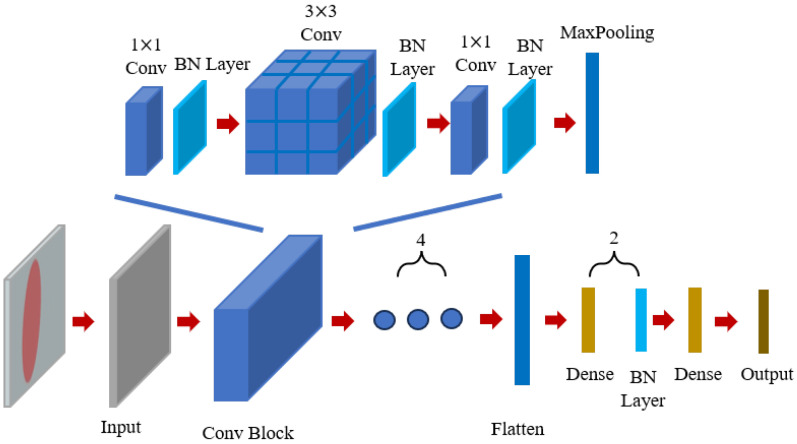
The diagram of the forward prediction convolutional neural network (CNN) structure. Each convolution block contains 3 convolutional layers, each with the same number of filters, and the sizes of the filters are 1 × 1, 3 × 3, and 1 × 1, respectively.

The framework platform we use to train the CNN was PyTorch (version 1.8.0), and we coded in Python 3.7.0 on Visual Studio Code (version 1.80). Since the dataset in this paper is in the form of structural images, it allows us to build a forward prediction network (FPN) based on a convolutional neural network (CNN). The structure of the forward prediction network designed in this paper is shown in Figure 3, consisting mainly of 4 convolutional blocks, 1 flatten layer, and 3 fully connected layers. Each convolutional block contains 3 convolutional layers, with each layer having the same number of filters, with filter sizes of 1 × 1, 3 × 3, and 1 × 1, respectively, and activated by the ReLU function. The ReLU function, also known as the modified linear unit, is an activation function commonly used in artificial neural networks, usually referring to the nonlinear function represented by the slope function and its variants. Batch normalization (BN) is applied after each convolutional layer, followed by a max-pooling layer for output. In this forward prediction network for metasurfaces, the number of filters in the convolutional layers of the 4 convolutional blocks is [32, 64, 128, 256], respectively, jointly transforming the (64, 64, 1)-sized metasurface structural image into a (8, 8, 256)-sized feature map. The final part of the network consists of a flatten layer and two fully connected layers, where the flatten layer unfolds all parameters of the (8, 8, 256)-sized feature map to obtain a feature space containing all feature map data, with a length of 16,384. The three fully connected layers further learn from the feature space. Ultimately, the input to the forward prediction network is a 64 × 64-pixel single-channel binarized image, and it outputs a vector of length 202. The first 101 data points of this vector represent the transmission spectrum $T_{x}$ of the current input structural image to the forward prediction network, while the remaining 101 data points represent $T_{y}$.

The training of a neural network requires determining the loss function and the optimizer. The output of the metasurface forward prediction network is the transmission spectra of the metasurface structure for polarized light in two directions. Essentially, it is a regression analysis of curves. Therefore, the mean square error $L_{\mathrm{pred}}$ is used as the loss function as follows:

(1) $$L_{\mathrm{pred}}=\frac{\sum_{i=1}^{N}{\left(T_{\mathrm{pred}}^{\left(i\right)}-T_{\mathrm{sim}}^{\left(i\right)}\right)}^{2}}{N}$$

In the formula, $T_{\mathrm{pred}}$ and $T_{\mathrm{sim}}$ are the transmittances of the predicted transmission spectrum and the FDTD-simulated transmission spectrum of the metasurface structure at the *i*-th sampling wavelength, respectively. The optimizer (i.e., the optimization algorithm) defines the learning rate, the number of training epochs, and the batch size of the training process. In this paper, the Adam optimizer is selected for training the metasurface forward prediction network. We have determined the initial learning rate and the decay principle: the initial learning rate is set to 0.001. During the training process, the change in the loss function $L_{\mathrm{pred}}$ is monitored. If the model performance does not improve after 10 consecutive training epochs, the learning rate is reduced by half. In addition, to prevent overfitting, an early-stopping mechanism is also introduced. When the loss function of the model does not improve after 20 consecutive training epochs, it is considered that the model has converged, and the training is immediately terminated. The difference between the predicted transmission spectrum and the simulated transmission spectrum is measured. $L_{\mathrm{pred}}$ is defined in the form of Equation (1).

**Figure 4:**
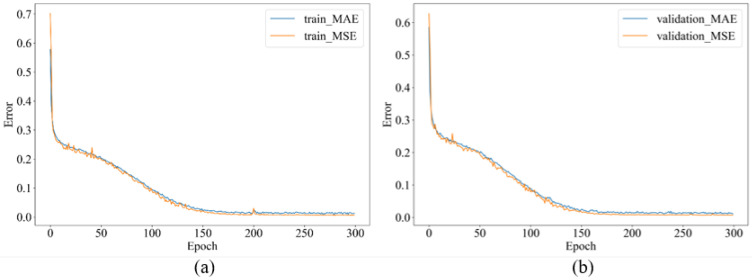
Mean squared error (MSE) as a function of training epochs. (**a**) Training set; (**b**) validation set.

In our dataset processing, we linearly sampled the dataset according to the metasurface’s structural dimensions to ensure the generalization ability of the neural network. To prevent it from falling into local optima, we shuffled the dataset’s order before splitting it into training, testing, and validation sets, with proportions of 70%, 15%, and 15%, respectively. Each training round involved batching the training set data in groups of 64 (BatchSize = 64) into the network. We recorded the mean square error (MSE) of the training and validation sets after each round of training. Figure 4 illustrates the changes in MSE and root mean square error (RMSE) over training rounds. After 300 rounds of training, the forward prediction network stabilizes, with the final results showing MSEs of $5\times 10^{-3}$ and $7\times 10^{-3}$ for the training and validation sets, respectively. It can be observed that the forward prediction network, using images as input, achieves high network accuracy with only around 2200 sets of data for training.

**Figure 5:**
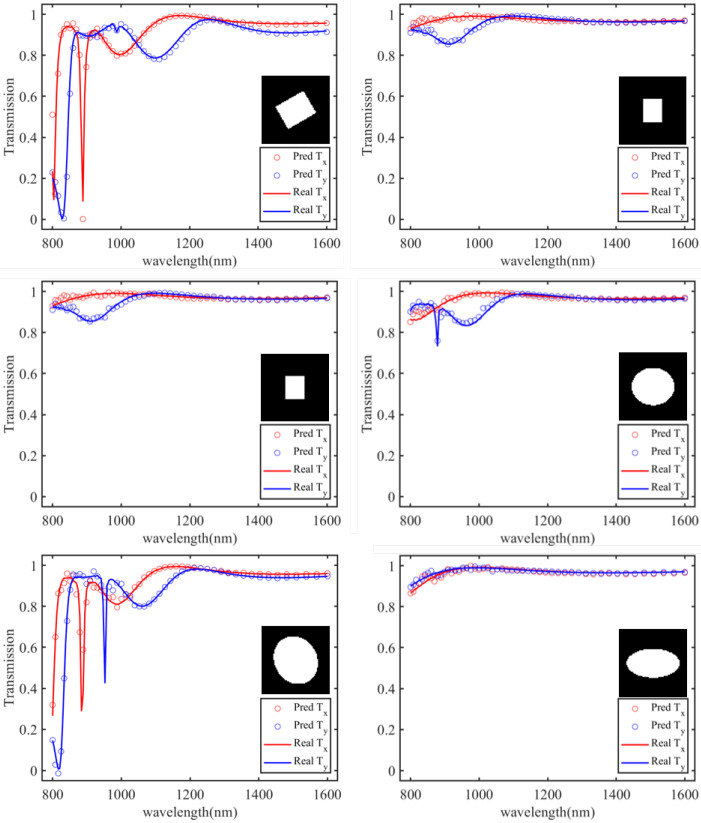
Experimental results for the transmission spectrum prediction part of the metasurface forward prediction network. The solid lines in the figure represent the actual transmission spectra obtained through simulation for the meta-atoms, and the dots indicate the predicted transmission spectra calculated by the forward prediction neural network. Red is used to represent the transmission spectra for light polarized in one direction, while blue represents the transmission spectra for light polarized in the orthogonal direction.

To assess the predictive capability of the forward prediction neural network, we conducted prediction experiments on the transmission spectra of metasurface structures in the test set. We compared the prediction results with simulation results and calculated the mean square error (MSE). Partial experimental results are shown in Figure 5. In the figures, solid lines represent the actual transmission spectra obtained through simulation for meta-atoms, while dots represent the predicted transmission spectra computed by the forward prediction neural network, depicted in red for $T_{x}$ and blue for $T_{y}$. To better observe the degree of fit between the predicted and actual transmission spectra, the predicted transmission spectra are plotted with data points at intervals of 51 points only, with corresponding meta-atoms displayed in the figure. It can be observed that the predicted transmission spectra closely match the actual transmission spectra, with a high degree of fit. They almost perfectly coincide in the high-transmission regions at longer wavelengths, and effectively capture the resonance points of each structure in the shorter-wavelength resonance regions. Similarly, the deviation between the predicted and actual transmission spectra is quantitatively represented by the MSE. On the test set of 470 data points, the MSE of the forward prediction network is approximately $1\times 10^{-3}$, meeting the design requirements and the precision demands of simulation software.

## 3. Results and Discussion

Through the aforementioned research, we have completed the design of the forward prediction network and validated its effectiveness. In fact, in many scenarios, metasurface design extends beyond individual surface structures. To verify the good generalization ability of our designed network, we employed it for predicting the transmission spectra of meta-atoms with high-quality resonance modes, based on transfer learning. This enables its wider application. In this study, we introduced a method to obtain high-quality-factor resonance meta-atoms by breaking the in-plane symmetry, specifically by applying a rotation to the double-elliptical structure with the same size and opposite direction.

**Figure 6:**
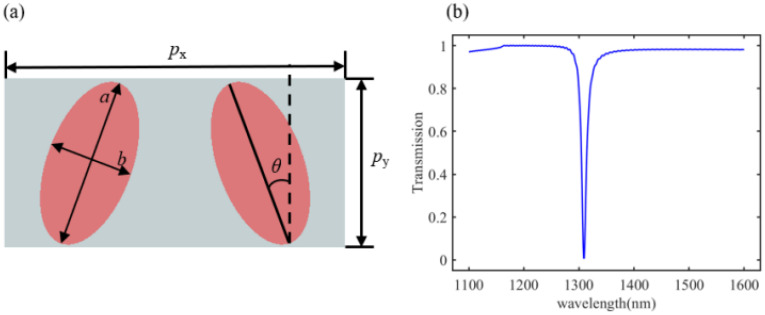
Resonance wavelength simulation results. (**a**) Transmission peaks vary with the short axis *b* at $\theta =21^{\circ }$; (**b**) transmission peaks vary with the twist angle when b=200 nm.

Figure 6 illustrates the double-elliptical structure designed in our laboratory and its simulated transmission spectra. This meta-atom can be viewed as a composite unit obtained by combining the aforementioned single-elliptical cylinder structures. Here, $p_{x}=800$ nm and $p_{y}=400$ nm represent the periodicities in the *x* and *y* directions of the double-elliptical unit, *a* and *b* represent the major and minor axes of the elliptical cylinders, and θ represents the rotation angle.

**Figure 7:**
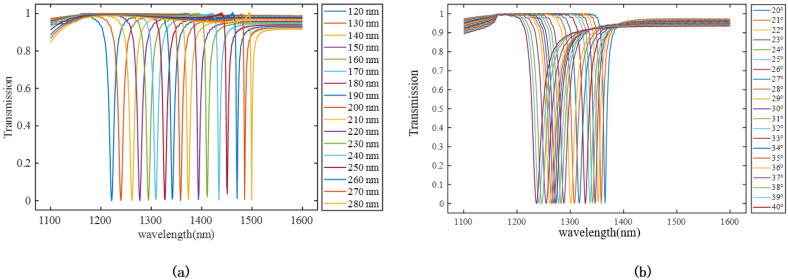
The structural symmetry was broken by adding equal-sized, opposite-direction twists to two elliptical cylinders, resulting in a meta-atom with a high quality factor, which allows for the observation of distinct resonance peaks in the near-infrared spectrum. (**a**) Schematic diagram of the designed dual-elliptical structure; (**b**) transmission spectrum with distinct resonance peaks.

Here, the variation in resonance peaks was simulated using FDTD Solutions. With the periodicities of the double-elliptical unit and the major axis of the elliptical cylinder set to $p_{x}=400$ nm, changes in the resonance wavelength were simulated by varying the lengths of the minor axis *b* and the rotation angle θ. The simulation results for the resonance wavelengths are illustrated in Figure 7.

From Figure 7, it can be observed that the aspect ratio, i.e., the ratio of the lengths of the major and minor axes, as well as the size of the rotation angle, significantly affects the resonance peak position of the meta-atom. Therefore, by varying the length of the minor axis and the rotation angle, we obtained a dataset of 1071 structures and their corresponding transmission spectra through parameter scanning. Similarly, by using a binarization method, we converted the structural images into binary images. Since the meta-atom images exhibit symmetry along the x-axis, we selected the left ellipse from symmetric structures as the input. The input image size was set to 64 × 64 pixels.

Due to the high similarity between this elliptical unit and the square pillar and elliptical cylinder structures in the previous dataset, we froze the parameters of the convolutional layers used to extract image features in the previously trained forward network. Only the fully connected layers used to establish the mapping from image features to spectral responses were trained. This transfer learning strategy helps address the data scarcity issue and enables more effective utilization of information in the existing dataset. By transferring knowledge from the pretrained model to the new task, we can build a model with good performance on relatively few data samples. This approach not only improves training efficiency but also enhances the generalization ability of the model to new tasks. To obtain more accurate resonance peak positions and peak values, the transmission spectra were computed only for the polarization direction and sampled at 501 points between 1100 nm and 1600 nm. The dataset was then input into the forward prediction network for training. After 500 rounds of training, the mean square error reached $3.95\times 10^{-3}$, demonstrating the strong predictive ability of the forward prediction network for composite unit structures.

**Figure 8:**
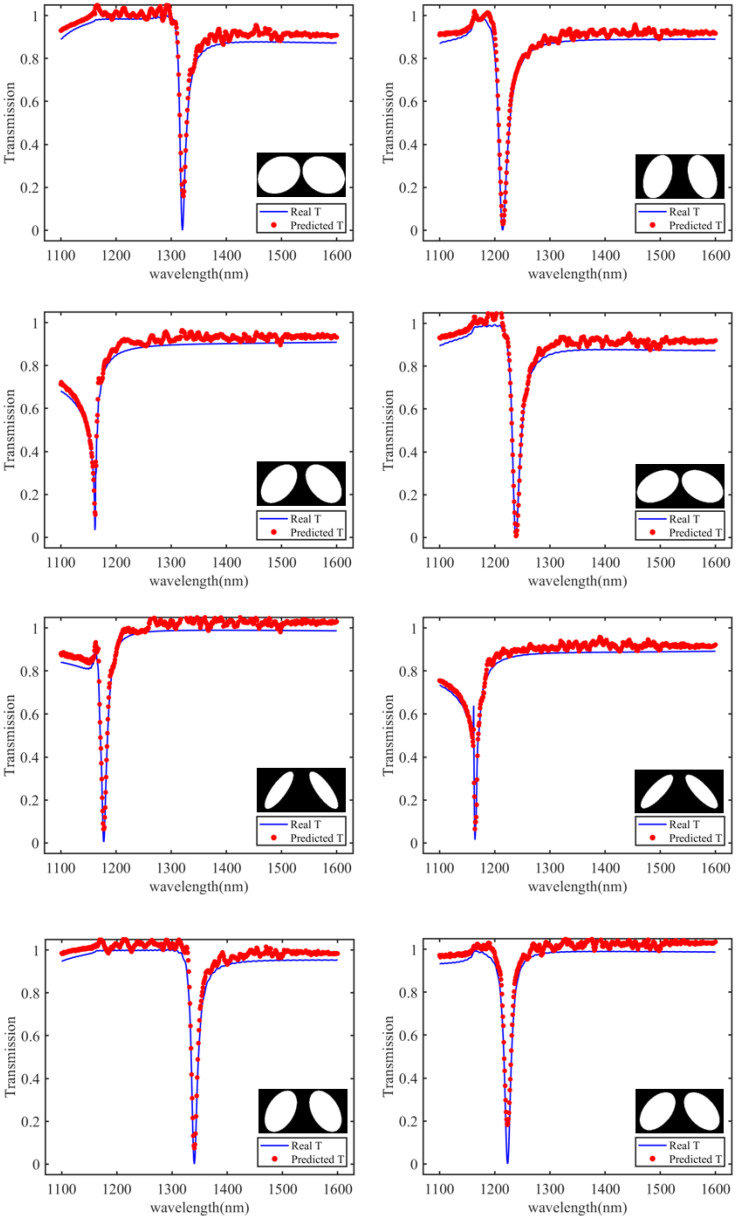
Prediction results of the FPN (forward prediction network) for the transmission peaks of the dual-elliptical meta-atom. The blue curve represents the simulation results, and the red dots indicate the predictions made by the neural network.

Figure 8 illustrates the predicted transmission spectra for the double-elliptical structure. For the 1071 sets of data, we calculated the relative error between the resonance peak wavelength and the Q value for the simulation and prediction results, respectively. For the resonant peak wavelength, the minimum relative error between the two can reach $6.2\times 10^{-4}$, while for the Q value, the minimum relative error between the two can reach $6.6\times 10^{-4}$. In fact, as to high Q metasurfaces, the wavelength of the resonance peak is often more important than the specific value of the Q value in practical applications, so that specific sensing and applications can be made. On this basis, we calculated the average relative error of the resonance peak wavelength, and the result is $1.89\times 10^{-2}$, which shows that the prediction is accurate. It can be seen that our designed network rapidly and accurately obtains the transmission spectra and resonance peak positions. Comparing the computation time between our designed forward prediction network and FDTD Solutions simulation software, FDTD Solutions took 3 h and 50 min to scan and collect data for 1071 sets, while the neural network required less than 1 s to predict the transmission spectra of any double-elliptical unit structure, greatly improving the efficiency of forward design.

The prediction of optical responses for complex structures is not addressed in this work. Addressing such prediction scenarios may necessitate more detailed investigations. The structural configurations covered in this work include elliptical, square, and circular geometries, as well as angle-varied configurations to control symmetry. Spectral prediction for more intricate structures may impose higher demands on dataset curation. Additionally, overlapping spectral phenomena in complex structures may require corresponding refinements in deep neural networks to ensure robust performance.

## 4. Conclusions

This study investigated a deep learning-based dielectric metasurface optimization design algorithm, starting from a dataset of meta-atoms. Initially, finite-difference time-domain simulations were employed to simulate the transmission spectra of two types of structures: square pillars and elliptical cylinders. The top-down views of the metasurface structures were used as structural data in the dataset. To enrich the diversity of the dataset, data augmentation techniques were applied. Based on the dataset comprising metasurface structural images and transmission spectra, a metasurface forward prediction convolutional neural network was designed. The network architecture and training strategy were optimized to achieve high prediction accuracy while reducing the number of network parameters and improving computational efficiency.

Only by incorporating an accurate and fast forward prediction network can the inverse design directly obtain a metasurface unit structure that meets the requirements through the optical response. Therefore, the fast and high-precision metasurface forward prediction network designed in this paper has practical application value and can be directly used in the design of metasurfaces in the future.

## Data Availability

The original contributions presented in the study are included in the article, further inquiries can be directed to the corresponding authors.
